# The Native Hawaiian Insect Microbiome Initiative: A Critical Perspective for Hawaiian Insect Evolution

**DOI:** 10.3390/insects8040130

**Published:** 2017-12-19

**Authors:** Kirsten E. Poff, Heather Stever, Jonathan B. Reil, Priscilla Seabourn, Alexander J. Ching, Sayaka Aoki, Mitchel Logan, Jennifer R. Michalski, Jessika Santamaria, Jesse W. Adams, Jesse A. Eiben, Joanne Y. Yew, Curtis P. Ewing, Karl N. Magnacca, Gordon M. Bennett

**Affiliations:** 1 Department of Plant and Environmental Protections Sciences, University of Hawaii at Manoa, 2500 Campus Rd., Honolulu, HI 96822, USA; kepoff@hawaii.edu (K.E.P.); hstever@hawaii.edu (H.S.); jbreil@hawaii.edu (J.B.R.); pseabour@hawaii.edu (P.S.); aching2@hawaii.edu (A.J.C.); sayakaa@hawaii.edu (S.A.); loganm8@hawaii.edu (M.L.); jrmichal@hawaii.edu (J.R.M.); jsantama@hawaii.edu (J.S.); cpe1@hawaii.edu (C.P.E.); 2 C-MAIKI Consortium, University of Hawaii at Manoa, 1991 East-West Rd., Honolulu, HI 96822, USA; jyew@hawaii.edu; 3 Department of Botany, University of Hawaii at Manoa, 2500 Campus Rd., Honolulu, HI 96822, USA; jwadams@hawaii.edu; 4 College of Agriculture, Forestry and Natural Resource Management, University of Hawaii at Hilo, 200 W Kawili St., Hilo, HI 96720, USA; eiben@hawaii.edu; 5 Pacific Biosciences Research Center, University of Hawaii at Manoa, 1993 East West Road, Honolulu, HI 96822, USA; 6 Bernice Pauahi Bishop Museum, 1525 Bernice St., Honolulu, HI 96822, USA; knm956@gmail.com

**Keywords:** insect-microbe interactions, microbial ecology, symbiosis, microbiome, Hawaiian insects, *Wolbachia*

## Abstract

Insects associate with a diversity of microbes that can shape host ecology and diversity by providing essential biological and adaptive services. For most insect groups, the evolutionary implications of host–microbe interactions remain poorly understood. Geographically discrete areas with high biodiversity offer powerful, simplified model systems to better understand insect–microbe interactions. Hawaii boasts a diverse endemic insect fauna (~6000 species) characterized by spectacular adaptive radiations. Despite this, little is known about the role of bacteria in shaping this diversity. To address this knowledge gap, we inaugurate the Native Hawaiian Insect Microbiome Initiative (NHIMI). The NHIMI is an effort intended to develop a framework for informing evolutionary and biological studies in Hawaii. To initiate this effort, we have sequenced the bacterial microbiomes of thirteen species representing iconic, endemic Hawaiian insect groups. Our results show that native Hawaiian insects associate with a diversity of bacteria that exhibit a wide phylogenetic breadth. Several groups show predictable associations with obligate microbes that permit diet specialization. Others exhibit unique ecological transitions that are correlated with shifts in their microbiomes (e.g., transition to carrion feeding from plant-feeding in *Nysius wekiuicola*). Finally, some groups, such as the Hawaiian *Drosophila*, have relatively diverse microbiomes with a conserved core of bacterial taxa across multiple species and islands.

## 1. Introduction

All complex multicellular life evolved either in alliance with, or in defense from, microbes, which have dominated Earth for billions of years [1]. In order to fully understand organismal function, diversity, and evolution, microbial interactions should be an essential consideration. Recently, a large body of literature has revealed that bacterial symbionts shape insect ecology and evolution by providing a range of environmental services, or by manipulating host reproduction [2,3]. Although it is well understood that microbial symbionts played fundamental roles in the diversification of some of the largest insect orders (e.g., the plant-sap feeding Hemiptera), their roles in shaping species-level diversity in most insect groups remain relatively unknown [4,5,6,7]. Recent advances in molecular sequencing technologies provide the ability to tackle these questions at unprecedented scales [8,9,10]. Despite this, efforts to comprehensively address questions regarding the role of microbes in insect ecology and evolution are hindered by the geographically widespread nature of insect populations and closely related species.

We propose to develop the Hawaiian Archipelago as an insect-microbiome model system to tackle fundamental questions in insect evolution that currently elude study. The Hawaiian Islands offer a simplified, closed ecosystem where the histories of habitat formation and organismal evolution can be completely understood [11,12]. Each island is ecologically diverse, including habitats that range from high altitude aeolian deserts to dense rainforests and tropical bogs. The Archipelago has formed linearly over a host-spot in the middle of the Pacific Ocean, where it has remained one of the most isolated landmasses for over 80 million years [13]. Furthermore, the ages of the current high islands and their constituent habitats are known with some precision, having all formed within the last five million years [12,14]. These geological features have generated a discrete, diverse, and replicated time-series of habitats. Thus, the archipelago is a long-held evolutionary model-system, informing theory of organismal adaptation and diversification over the last several decades [15,16]. Hawaii uniquely provides a natural evolutionary experiment with a developed theoretical framework to extend this system to address explicit questions of host–microbiome interactions.

The endemic Hawaiian insects comprise over 10,000 described species that diversified from a mere ~260 colonization events [17,18,19]. For example, the Hawaiian *Drosophila* radiated from a single arrival into approximately 1000 species. *Drosophila* species specialize on specific plant taxa, plant parts, and fungi for feeding, mating, and oviposition [20,21,22]. Many other native insects, such as those that feed on plant-sap (e.g., Hemiptera), diversified into hundreds of species while specializing on endemic host-plants that have divergent natural defenses and nutritional qualities [23,24]. In some of these groups, species have transitioned to novel habitats and diets that are rare, or unknown in their continental relatives. These include species that have adapted to extreme habitats such as caves, sub-alpine and arid regions, or to completely new diets [25]. Specific examples include the iconic wekiu bug and sister taxon (Hemiptera: Heteroptera: *Nysius wekiuicola* and *Ny. aa*) that have transitioned from plant feeding to alpine scavenging of dead insects capable of withstanding sub-freezing temperatures—an adaptation not found in any other members of this globally distributed genus [26,27]; the caterpillar (*Eupithecia* spp.) that switched from a pollen diet to carnivory; and, the drosophilid fly (*Scaptomyza* [*Titanochaeta*] spp.) that transitioned from detritivory to spider egg predation [28,29]. The discrete origins and knowable phylogenetic history of endemic Hawaiian lineages permits targeted questions of how host–microbiome interactions have evolved through time and influenced host evolution.

Given the intrinsic role of microbes in shaping insect ecology and diversity, there is surprisingly little known about their role in the diversification of Hawaiian insect fauna. Perhaps the best understood is in Hawaiian *Drosophila*. Species in this group are known to associate with a wide-range of fungi that may play important roles in host-plant use [30,31]. Recent studies in other systems suggest that insect lineages represented in Hawaii likely maintain complex associations with bacteria. Expectations from non-island model systems provide a baseline prediction for Hawaiian taxa. For example, *Drosophila* species harbor a gut microbiome that can influence host development and fecundity [7,32]. Others, including plant-sap feeding leafhoppers (Cicadellidae), planthoppers (Cixiidae and Delphacidae), and heteropterans, have obligate associations with microbes that provide essential nutrition to their hosts [8,33,34]. Although host–microbe interactions are well understood for some insects such as those in the Hemiptera [35], the extent to which microbes influence insect adaption and diversity more broadly remains poorly understood.

To gain a better understanding of how microbes may shape insect evolution, we are initiating the Native Hawaiian Insect Microbiome Initiative (NHIMI). The goal of NHIMI is to develop a framework for understanding how insect–microbiome interactions shape biological diversity. To introduce this effort, we describe—for the first time—the bacterial diversity of 13 endemic Hawaiian insect species (eight genera and seven families). Although this is an initial survey, our sampling is designed to address two principal questions: (i) What is the expected diversity of microbes associated with representative insects that have widely different ecologies? (ii) Do adaptations to novel niches correlate with transitions in insect microbiomes? Elucidating the role of the host-associated microbiomes in the diversification of Hawaiian insects will help to modernize this long-held evolutionary model system to continue informing evolutionary theory well into the future.

## 2. Materials and Methods

### 2.1. Sample Processing and Sequencing

Insect specimens were collected from across the Hawaiian Islands (see Table 1 for localities). Species were identified by contributing authors with available taxonomic resources. In general, species were sequenced in triplicate except for some that were obtained from other collaborators (e.g., *Drosophila* hindgut dissections for three species provided by J. Yew; see Table 1). All field-collected specimens were stored in 100% EtOH at −20 °C immediately after collection. Sequenced samples were subsequently washed three times in 100% EtOH, except for the *Drosophila* gut dissections. To increase DNA yield from internal tissues, samples were manually macerated with a sterile pestle and homogenized for 10 min with 0.7 mm garnet beads. DNA was then extracted using a DNeasy PowerSoil Kit (Qiagen, Hilden, Germany). A final negative control following extraction and sequencing protocols was included to ensure clean sample processing and to test for kit contamination. Purified DNA was quantified with a Qubit 3.0 fluorometer (ThermoFisher, Waltham, MA, USA). Library preparation and genomic sequencing were performed by SeqMatic (Fremont, CA, USA) using a standardized protocol. PCR amplification targeting the meta-barcoding V4 region of bacterial 16SrRNA was achieved by the primer pair 515F (5′-GTGYCAGCMGCCGCGGTAA-3′) and 806R (5′-GGACTACNVGGGTWTCTAAT-3′) [36]. The thermocycling profile included an initial hold for 3 min at 94 °C followed by 36 cycles of: denaturation of 45 s at 94 °C, annealing at 50 °C for 60 s, and extension at 72 °C for 10 min. Libraries were sequenced with an Illumina MiSeq for paired-end reads (2 × 150 base pairs).

### 2.2. Bacterial Community Analyses

**Figure 1:**
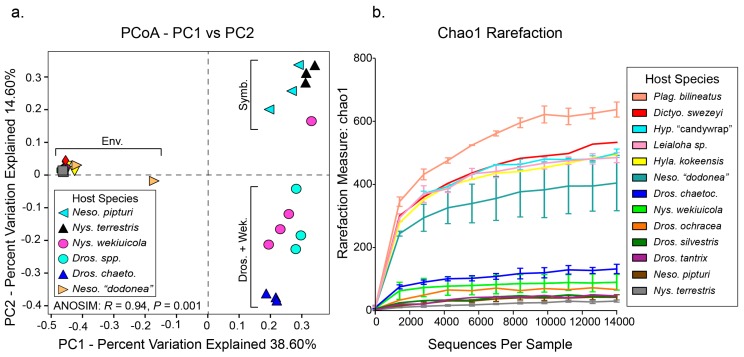
(**a**) PCoA of bacterial communities by insect sample based on unweighted unifrac distances. Samples that are stacked or tightly clustered are omitted from the inset legend. Clusters used in the ANOSIM analyses are labeled with brackets (Env. = Environmental, Symb. = intracellular symbiont associated, Dros. + Wek. = *Drosophila* spp. *Nysius wekiuicola*). (**b**) Rarefaction curves with Chao1 diversity indices, indicating insect microbiome sampling depth and saturation in this study. Legend illustrates host insect species identity.

**Figure 2:**
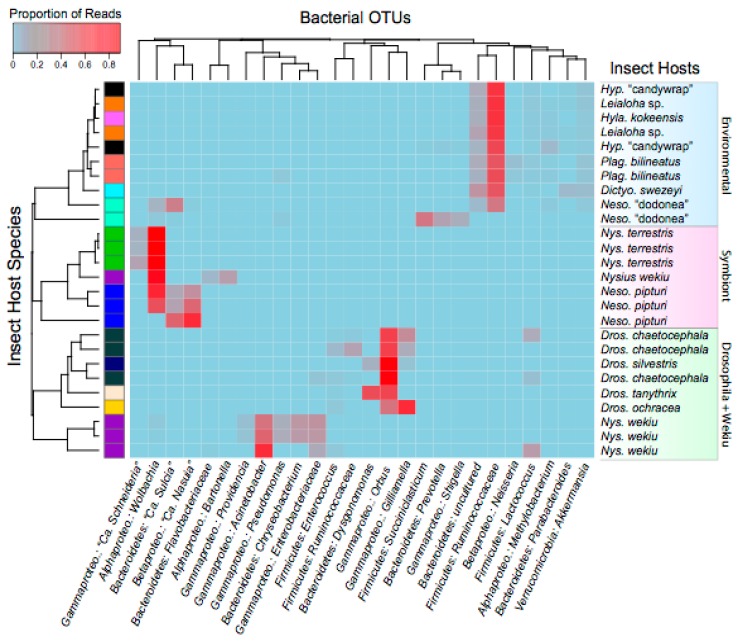
Heatmap showing bacterial taxa distributed across sequenced insect specimens. Bacterial OTUs that comprise less than 5% of total reads are excluded. Cell values are calculated proportionately across rows and dendrograms estimated with Bray–Curtis dissimilarity index. Clusters shown on the right side in shaded boxes (e.g., Environmental, Symbiont, etc.,) correspond to those also shown Figure 1a (ANOSIM: R = 0.94, *p* = 0.001). Some bacterial taxonomic names (e.g., *Betaproteo*. = *Betaproteobacteria*) have been abbreviated.

The FastX-Toolkit was used to filter low quality sequences (settings: fastq_quality_trimmer, -Q 33, -t 20, -l 130) [37]. At this step, two low quality samples were eliminated from the dataset. Sequences were then merged using PEAR V0.9.6 (settings: default) and sequences with ambiguous base-calls removed with prinseq-lite v0.20 (settings: default) [38]. Finally, chimera sequences were removed with VSEARCH using UCHIME (settings: default) [39,40]. Operational Taxonomic Unit (OTU) clustering was performed using UCLUST on MacQIIME V1.9.1 (*pick_open_reference_otus*.*py*) and taxonomic assignment performed at 97% against the SILVA database [41,42]. These data were used to determine alpha diversity, beta diversity indices, and clustering of species based community similarity (*core_diversity_analyses.py*) [41]. Sample reads were rarefied to 14,500 reads, which excluded three additional low-quality samples (see Table 1 and Table 2). OTU saturation was evaluated with rarefaction curves using chao1 richness estimate. Shannon Diversity and Pielou’s Evenness Indices were calculated for each sample with the vegan package in R V3.3.3 [43,44]. A two-sample *t*-test with non-parametric Monte Carlo permutations (*n* = 999) and Bonferroni correction was used to test for statistically significant differences in alpha diversity between species with biological replicates (QIIME script: *compare_alpha_diversity.py*). Samples represented by a single individual or only two replicates were removed. A two-dimensional principal coordinate analysis (PCoA; *make_2D_plots.py*) was conducted from unweighted unifrac distances obtained from core diversity analyses. Insect hosts clustered into three groups that include (1) an intracellular symbiont dominated microbiota group (e.g., obligate bacterial and facultative symbionts found in sap-feeding leafhoppers and seed bugs), (2) Drosophila + Wekiu bug (*Drosophila* spp. and *Nysius wekiuicola*), and an environmentally diverse microbiota group (e.g., *Plagithmysus bilineatus*, *Hyposmocoma* sp., *Leialoha* sp., etc.,) (see Figure 1 and Figure 2). In order to determine if these clusters are significantly dissimilar, an analysis of similarity (ANOSIM) was performed in QIIME with 999 permutations (*compare_categories.py*). Finally, to further illustrate the most abundant bacterial taxa and community relationships across host species a heatmap and dendrogram was generated with Bray–Curtis dissimilarity index and bacterial OTUs representing <5 reads removed in R V3.3.3 [43,44,45].

### 2.3. Data Availability

Bacterial 16S reads for each sample were submitted to GenBank Single Read Archive (SRA) database under accession numbers SAMN07806952-SAMN07806982.

## 3. Results and Discussion

**Figure 3:**
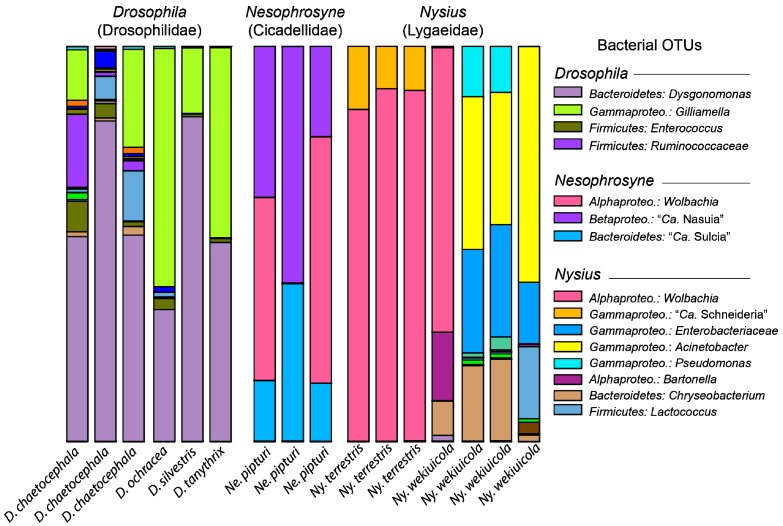
Relative abundance of bacterial groups observed in insects with different feeding behaviors. Different colors represent various bacterial Operational Taxonomic Units (OTUs) present in the corresponding samples. Note: Illustrative colors are distinct to each insect host microbiome community and only the most abundant bacterial taxa are included in the legend. Some taxonomic names (e.g., *Betaproteo*. = *Betaproteobacteria*) have been abbreviated.

The bacterial microbiomes of select endemic Hawaiian insect species were surveyed to provide baseline understanding of their community diversity and potential influence on host ecology and evolution. Insect species were selected to explicitly investigate (a) the microbial diversity associated with iconic Hawaiian host insects, and (b) the microbiome communities of hosts that have experienced novel niche transitions. Following read quality filtering and a stringent rarefaction threshold, we analyzed a total of 26 individuals in 13 endemic Hawaiian species (five orders and seven families; see Table 1). Broadly, our results reveal that Hawaiian insects harbor a wide diversity of bacterial lineages, comprising over 10 bacterial phyla (see Table S1). Several species have bacterial communities that appear to be distinct from those found in other host species sampled in our study (see Figure 1 and Figure 2; e.g., *Drosophila* spp., *Nysius* spp., and *Nesophrosyne* spp.). In the case of *Drosophila*, host insects appear to share members of their bacterial communities across replicates, distinct species, and geographic locations (Figure 1, Figure 2 and Figure 3). In other taxa, communities also appear to be related to insect habitat and diet (Figure 1, Figure 2 and Figure 3). For example, the plant-sap feeding insects, *Nysius terrestris* (which feed on seeds in addition to sap) and *Nesophrosyne pipturi*, both maintain obligate symbionts that provide essential nutrition absent in their plant-based diets (discussed further below) [46,47]. In addition, sap-feeders such as *Ny. terrestris* and *Ne. pipturi* had lower overall bacterial diversity than other insects with different feeding behaviors such as *Ny. wekiuicola* and *Drosophila* (see Shannon Indices in Table 2). However, we note that alpha diversity indices are not significantly different (*p* > 0.05) between samples at the species-level. This result is expected since our sampling was not exhaustive, lacking sampling breadth and suitable replication for each taxon or species. Nevertheless, statistical analysis of clustering across all samples is highly significant (ANOSIM: R = 0.94, *p* = 0.001; see Figure 1 and Figure 2), indicating that native Hawaiian insects may have emergent microbiome properties. Species appear to cluster into categories corresponding to host-level relationships with obligate and facultative intracellular symbionts, host transitions to novel trophic levels (e.g., carrion feeding from plant feeding in *Ny. wekiuicola*), insect host identity (*Drosophila* spp.), and insects with potentially diverse environmentally assembled microbiomes (e.g., *Hyposmocoma* spp. and *Plagithmysus* spp.; see discussion below).

Many insect groups are known to have diverse bacterial microbiomes that reside in the gut, but that also contain members occurring in other tissues (e.g., specialized bacteriome organs and gonads) [48,49,50]. These microbes can provide a range of services that affect host development, fecundity, pathogen susceptibility, and the breakdown of environmental compounds [51,52,53,54,55]. Our results reveal that some Hawaiian insect species have diverse microbiomes that are not differentiated across species spanning higher taxonomic rankings (e.g., environmental cluster, see Figure 1 and Figure 2) that include Delphacidae planthoppers, Cosmopterigidae: *Hyposmocoma* moths, among others). Generally, these species have broad ecological niches and feeding habits such as wood-boring and herbivory (e.g., Cerambycidae: *Plagithmysus bilineatus*) or lichenivorous (e.g., *Hyposmocoma* “sp. candywrap-case”) (Table 1). The delphacid planthoppers are known to harbor obligate fungal symbionts that aid in plant-sap feeding that would not be detected in our bacterial barcoding approach, possibly leading to increased sampling of lower abundance bacteria (e.g., *Leialoha* sp. and *Dictyophorodelphax swezeyi*; see [33]). Our analysis further reveals that these insect groups have complex, possibly incidental species assembled from environmental interactions associated with feeding. However, it is notable that some insects, such as the herbivorous cerambycid beetles, can have relatively cellulose-rich diets. Intriguingly, they were found to maintain bacteria in the Ruminococcaceae family (Firmicutes) in relatively high abundance, which are capable of degrading cellulose-based substrates (Figure 2) [53]. Otherwise, the functional roles of the microbiomes within these insect groups are currently unknown.

Among insects, the potential roles of the gut microbiome are perhaps best studied in *Drosophila*, which are an important model system for microbiome and genetic studies [56,57,58]. *Drosophila* arrived on Hawaii ~25 million years ago and subsequently diversified to feed and oviposit on specific plant parts in over 40 Hawaiian endemic plant families [12,59,60,61]. Species in this group are detritivorous, feeding on decaying plant material in a microbe-rich environment. Although our sampling here is limited to four species (of almost 1000 species), our preliminary survey found evidence that the Hawaiian *Drosophila* microbiome maintains a conserved set of bacterial taxa. The identities of the most abundant bacterial species across *Drosophila* samples are similar regardless of whether guts or whole-bodies were sampled (Figure 1, Figure 2 and Figure 3, Table 2). Community conservation is observed among replicates for *Drosophila chaetocephala*, and across multiple species that have different host-plants and are restricted to different islands (Table 1). The consistent, relatively high abundance members of this microbiome include the Orbaceae family and the genus *Dysgonomonas* (*Bacteroidetes*) (Figure 2 and Figure 3). These bacterial taxa have been identified as core members of the gut microbiomes in non-Hawaiian *Drosophila* species across the globe and also in other insects, including termites and honeybees [62,63,64,65,66]. Although the importance of these microbes in Hawaiian *Drosophila* remain unknown, given their widely conserved nature it is plausible that they play important roles in shaping host ecological adaptations. It would be illustrative for future studies to investigate microbiome community structure across Hawaiian *Drosophila* phylogenetic diversity, geographic ranges, and ecological associations. Sampling should further include food substrates to distinguish their gut microbiota from the environment. Finally, the Hawaiian *Drosophila* feed and oviposit on rotting and decaying plant material that are rich in fungi and yeasts. Surveys of Hawaiian *Drosophila* microbiome communities should include these microbial groups as they likely have important implications for host ecology and evolution [30].

As expected, the sampled plant-sap feeding insect taxa harbor predictable and conserved microbiome communities. In general, these communities comprise known obligate symbionts that provide essential functions and that are required for host reproduction. For example, *Ne. pipturi* and *Ny. terrestris* maintain microbiome communities that include previously identified obligate bacterial symbionts (Figure 2 and Figure 3) [46,47]. These species also have reduced complexity in their microbiome communities, perhaps due to either (i) their relatively sterile feeding habit that reduces their contact with environmental microbes, or (ii) overrepresentation of intracellular obligate and facultative symbionts (see Figure 1 and Figure 2 and see also discussion below). In the case of *Ne. pipturi*, a high abundance of reads grouped with the obligate symbiont species, “*Candidatus* Sulcia muelleri*”* (*Bacteroidetes*) and “*Ca*. Nasuia deltocephalinicola” (*Betaproteobacteria*) (20–30% of relative bacterial diversity, respectively), which are derived from ancient symbioses in the Auchenorrhyncha [66]. Genomic work has shown that these obligate symbionts are required for the synthesis of essential amino acids that are in low abundance in their hosts’ plant-sap diets [46,67,68,69]. Thus, it is expected that these symbionts would be found broadly across other related endemic Hawaiian Auchenorrhyncha, including some planthopper families (e.g., Fulgoroidea: Cixiidae) that also rely on nutrient limited diets [70].

The existence of obligate bacterial symbionts among the microbiomes of some insect groups provides a predictable null expectation for host–symbiont interactions under normal conditions. This natural experimental framework provides a testable hypothesis regarding how microbiome communities influence—or are influenced by—host transitions to novel niches, particularly if community membership is altered. In our study, we found such evidence that an obligate symbiont replacement may be occurring in at least one *Nesophrosyne* species. While the microbiome of *Ne. pipturi* was consistently observed to contain high relative abundances of the obligate symbionts (“*Ca*. Sulcia” and “*Ca*. Nasuia”), the undescribed *Nesophrosyne* “dodonea” species appears to occasionally lack “*Ca*. Nasuia” (Figure 2). Our recent total meta-genomic sequencing of this host also supports this finding (Bennett unpub. data). “*Ca*. Nasuia” is known to have been replaced with some frequency throughout the Cicadomorpha (e.g., sharpshooter leafhoppers, cicadas, spittlebugs) [71,72]. Although the cause for a potential disruption of this symbiosis is unclear, it is notable that *Ne.* “dodonea” tends to live in an arid and hot habitat with many non-native organisms, which is unusual for members of this genus [24].

One clear example of a microbiome shift is found in the wekiu bug (Heteroptera: *Nysius wekiuicola*). *Nysius wekiuicola* transitioned from plant feeding at low elevations to scavenging carrion on the sub-alpine summit of Mauna Kea (~4000 m)—an adaptation not known to occur in any other species in the genus [26,27]. Members of the *Nysius* genus harbor an obligate symbiont, “*Ca.* Schneideria nysicola” (*Gammaproteobacteria*). Although no genome for this symbiont is currently available to understand its metabolic contributions, it is thought to nutritionally supplement its hosts’ plant-based diet [47]. As expected, “*Ca.* Schneideria” was found in relatively high abundance in the endemic plant-feeding species, *Ny. terrestris* (average of ~12.6% of bacterial diversity in infected individuals; Figure 2 and Figure 3). It is likely conserved broadly across the endemic Hawaiian species in the genus. In contrast, *Ny. wekiuicola* appears to no longer harbor “*Ca.* Schneideria” in correlation with its extreme ecological and dietary adaptations. Shifting to carrion feeding likely provides a more complete nutritional profile (e.g., balanced essential amino acids and other nutrients), belying the requirement to maintain populations of symbiotic bacteria [73,74]. Our results further show that *Ny. wekiuicola* has a diverse and distinct microbiome than *Ny. terrestris* (Figure 1 and Figure 3; see also Shannon Indices in Table 2). Although the functional role of these microbes remains uncertain, it is possible that *Ny. wekiuicola*’s enriched bacterial community is at least partially derived from its diet of decomposing insects exposed to environmental microbes.

Finally, since we generally sampled whole insect bodies (i.e., all organs), we were able to detect other facultative and parasitic bacteria that do not necessarily reside in the gut. For example, several Hawaiian insect species are highly infected with *Wolbachia*, which includes several sap-feeding insects sampled in this study (*Nesophrosyne* spp. and *Nysius* spp.; average of 33% of relative bacterial diversity when infected). However, *Wolbachia* infection was not found across all species’ replicates, which is expected since parasitic and facultative symbionts often do not infect every individual in a population [2,75]. *Wolbachia* is one of the most widespread insect symbionts; it infects up to 60% of all arthropod species, including endemic Hawaiian insect lineages (e.g., *Nesophrosyne* and *Drosophila*) and is known to have parasitic or mutualistic interactions with its hosts [75,76,77,78,79]. Whole-body surveys of insect microbiomes provide an opportunity to screen for non-gut associated pathogenic and facultative microbes. However, we caution that facultative symbionts at high titers may dominate microbiome communities and cause other low abundance bacterial taxa in the gut to be under-sampled (e.g., see *Ny. wekiuicola* in Figure 1 and Figure 3). Adequate sequencing depth is required to thoroughly investigate the complete microbiome profile of individual insects. We further caution that insect specimens infected with *Wolbachia* may cluster with others that are also infected, but that have a distinct and under sampled microbiome as in the case of one *Ny. wekiuicola* replicate (see Figure 1, Figure 2 and Figure 3). Thus, it is critically important to obtain suitable biological replication to thoroughly understand host-associated microbiomes.

## 4. Conclusions

The aim of the Native Hawaiian Insect Microbiome Initiative (NHIMI) is to develop a framework to understand the synergies between host-associated microbiomes and insect species diversity. The Hawaiian Islands provide a natural platform for more precise investigation of the role host–microbe interactions play in shaping insect ecology and evolution. Linking these aspects allow for the opportunity to better understand the mechanisms that underlie adaptive diversification in animals more broadly. Even though our preliminary study includes a limited number of endemic species, our results do provide a set of baseline predictions for several of the largest and most iconic insect radiations on Hawaii. We emphasize that broad scale microbiome community analyses without attention to the identity of particular bacterial species may miss important biological aspects of host–microbe associations in Hawaiian insects. For example, although the obligate symbiont in *Ny. terrestris,* “*Ca.* Schneideria”, is fundamental to host fitness, it only represents ~12.6% of the total microbiome community abundance, far less than *Wolbachia* when present. The related *Ny. wekiuicola* appears to have lost “*Ca.* Schneideria” completely in association with its adaptation to a novel—and dramatically different—ecological niche. Similarly, the Hawaiian *Drosophila* maintain a fairly diverse microbiome, but they contain several species in lower abundance that are uniquely shared between Hawaiian *Drosophila*, and also with *Drosophila* species worldwide. Thus, establishing a baseline understanding for the emergent properties of host-associated microbiomes in Hawaiian insects is critical to elucidating conserved symbiotic interactions. These types of symbioses are likely to have important implications for host ecology and evolution.

The insights produced by this study (derived from a graduate student class) could be easily expanded by a concerted effort from the Hawaiian entomological research community. Our immediate goals are to (a) increase host taxonomic sampling to encompass all major insect lineages and species diversity, and (b) survey the other microbial groups that include the archaea, fungi, and even viruses [80,81]. It is our long-term goal to encourage other entomological researchers to collaboratively consider the importance of microbes in shaping the biology of their study organisms. We view that incorporating a microbiome perspective is essential to not only fully understanding insect evolution, but also to maintaining Hawaii as a cutting-edge model system for guiding evolutionary theory more broadly.

## Figures and Tables

**Table 1:** Taxon sampling for 16S bacterial metabarcoding.

Family	Species	Collection Location	Feeding Behavior	Replicates
Cerambycidae	*Plagithmysus bilineatus*	Hawaii Island, HI, Upper Wailuku	Herbivorous	3 ^2^
Cicadellidae	*Nesophrosyne pipturi*	Oahu, HI, Manoa Cliff Tr.	Sap-feeding	3
Cicadellidae	*Nesophrosyne* “dodonea” ^1^	Oahu, HI, Diamond Head	Sap-feeding	2
Lygaeidae	*Nysius terrestris*	Hawaii Island, HI, Mauna Kea	Sap/seed-feeding	3
Lygaeidae	*Nysius wekiuicola*	Hawaii Island, HI, Mauna Kea	Carnivorous	5 ^2^
Delphacidae	*Dictyophorodelphax swezeyi*	Kauai, HI, Kokee State Park	Sap/seed-feeding	1
Delphacidae	*Leialoha* sp. ^1^	Oahu, HI, Kaala Road	Sap/seed-feeding	3 ^2^
Drosophilidae	*Drosophila chaetocephala*	Oahu, HI, Mt Kaala	Detritivorous	3
Drosophilidae	*Drosophila ochracea*	Hawaii Island, HI	Detritivorous	1 ^3^
Drosophilidae	*Drosophila silvestris*	Hawaii Island, HI	Detritivorous	1 ^3^
Drosophilidae	*Drosophila tanythrix*	Hawaii Island, HI	Detritivorous	1 ^3^
Cosmopterigidae	*Hyposmocoma* “sp. candywrap-case” ^1^	Oahu, HI, Palikea	Lichenivorous	3 ^2^
Colletidae	*Hylaeus kokeensis*	Kauai, HI	Flower Feeding	1
Colletidae	*Hylaeus kauaiensis*	Kauai, HI	Flower Feeding	1 ^2^

^1^ Species have not yet been formally described; ^2^ taxa were eliminated from analysis due to low quality sequencing output; ^3^ hindgut tissues.

**Table 2:** Bacterial 16S barcoding sequencing depth and quality.

Family	Seq ID	Species	Raw Reads	Merged Fragments	Shannon Diversity Index	Pielou’s Evenness Index
Cerambycidae	IM_001	*Plagithmysus bilineatus*	62,589	4067	N/A	N/A
	IM_002	*Plagithmysus bilineatus*	210,792	32,601	2.78	0.519
	IM_003	*Plagithmysus bilineatus*	279,336	48,751	2.92	0.528
Cicadellidae	IM_006	*Nesophrosyne pipturi*	281,608	28,096	1.19	0.498
	IM_007	*Nesophrosyne pipturi*	285,705	54,199	1.03	0.402
	IM_008	*Nesophrosyne pipturi*	408,688	37,665	1.08	0.437
	IM_064	*Nesophrosyne* “dodonea” ^1^	140,222	20,007	3.54	0.713
	IM_065	*Nesophrosyne* “dodonea” ^1^	275,866	40,540	2.32	0.462
Colletidae	IM_054	*Hylaeus kokeensis*	135,535	23,067	2.23	0.453
	IM_057	*Hylaeus kauaiensis*	80,603	10,664	N/A	N/A
Cosmopterigidae	IM_047	*Hyposmocoma* sp. ^1^ (candywrap-case)	68,231	7180	N/A	N/A
	IM_048	*Hyposmocoma* sp. ^1^ (candywrap-case)	332,269	52,954	2.40	0.448
	IM_049	*Hyposmocoma* sp. ^1^ (candywrap-case)	141,714	20,496	2.58	0.509
Delphacidae	IM_029	*Dictyophorodelphax swezeyi*	157,229	30,539	2.64	0.523
	IM_041	*Leialoha* sp. ^1^	180,872	28,045	2.39	0.465
	IM_042	*Leialoha* sp. ^1^	92,971	6127	N/A	N/A
	IM_043	*Leialoha* sp. ^1^	214,609	32,972	2.28	0.442
Drosophilidae	IM_044	*Drosophila chaetocephala*	205,429	30,495	2.66	0.652
	IM_045	*Drosophila chaetocephala*	222,912	37,809	1.45	0.378
	IM_046	*Drosophila chaetocephala*	208,202	29,385	2.33	0.620
	IM_050	*Drosophila ochracea*	193,518	32,766	1.57	0.524
	IM_051	*Drosophila silvestris*	251,611	58,065	1.18	0.416
	IM_052	*Drosophila tanythrix*	317,357	58,839	0.87	0.315
Lygaeidae	IM_015	*Nysius terrestris*	362,988	44,577	0.56	0.226
	IM_016	*Nysius terrestris*	421,244	33,881	0.45	0.230
	IM_017	*Nysius terrestris*	370,619	34,477	0.45	0.230
	IM_025	*Nysius wekiuicola*	365,402	36,829	0.92	0.331
	IM_059	*Nysius wekiuicola*	108,745	14,561	2.23	0.597
	IM_060	*Nysius wekiuicola*	121,857	19,236	2.26	0.604
	IM_062	*Nysius wekiuicola*	127,518	15,219	2.11	0.580
	IM_063	*Nysius wekiuicola*	108,665	10,776	N/A	N/A
Control	C-O	N/A	1297	263	N/A	N/A

^1^ Species have not been formally described.

## Supplementary Materials

The following are available online at www.mdpi.com/2075-4450/8/4/130/s1, Table S1: Complete Bacterial Operational Taxonomic Unit (OTU) table with full taxonomic names.

## References

[1] McFall-Ngai M., Hadfield M.G., Bosch T.C.G., Carey H.V., Domazet-Lošo T., Douglas A.E., Dubilier N., Eberl G., Fukami T., Gilbert S.F. (2013). Animals in a bacterial world, a new imperative for the life sciences. Proc. Natl. Acad. Sci. USA.

[2] Moran N.A., McCutcheon J.P., Nakabachi A. (2008). Genomics and evolution of heritable bacterial symbionts. Annu. Rev. Genet..

[3] Ferrari J., Vavre F. (2011). Bacterial symbionts in insects or the story of communities affecting communities. Philos. Trans. R. Soc. B.

[4] Douglas A.E. (1998). Nutritional interactions in insect-microbial symbioses: Aphids and their symbiotic bacteria Buchnera. Annu. Rev. Entomol..

[5] Ishikawa H., Bourtzis K., Miller T.A. (2003). Insect Symbiosis: An Introduction in Insect Symbiosis.

[6] Hurst C.J. (2016). The Mechanistic Benefits of Microbial Symbionts.

[7] Martinson V.G., Douglas A.E., Jaenike J. (2017). Community structure of the gut microbiota in sympatric species of wild Drosophila. Ecol. Lett..

[8] McCutcheon J.P., Moran N.A. (2012). Extreme genome reduction in symbiotic bacteria. Nat. Rev. Microbiol..

[9] Goodwin S., McPherson J.D., McCombie W.R. (2016). Coming of age: Ten years of next-generation sequencing technologies. Nat. Rev. Genet..

[10] Muir P., Li S., Lou S., Wang D., Spakowicz D.J., Salichos L., Zhang J., Weinstock G.M., Iaacs F., Rozowsky J. (2016). The real cost of sequencing: Scaling computation to keep pace with data generation. Genome Biol..

[11] Fleischer R.C., McIntosh C.E., Tarr C.L. (1998). Evolution on a volcanic conveyor belt: Using phylogeographic reconstructions and K–Ar-based ages of the Hawaiian Islands to estimate molecular evolutionary rates. Mol. Ecol..

[12] Price J.P., Clague D.A. (2002). How old is the Hawaiian biota? Geology and phylogeny suggest recent divergence. Proc. R. Soc. Lond. B Biol..

[13] Clague D.A., Sherrod D.R., Poland M., Takahashi T.J., Landowski C.M. (2014). Growth and degradation of Hawaiian volcanoes in Characteristics of Hawaiian Volcanoes. Geol. Surv. Prof. Pap..

[14] Alison K.E. (1994). A Natural History of the Hawaiian Islands: Selected Readings II.

[15] Carson H.L., Templeton A.R. (1984). Genetic revolutions in relation to speciation phenomena: The founding of new populations. Annu. Rev. Ecol. Syst..

[16] Shaw K.L., Gillespie R.G. (2016). Comparative phylogeography of oceanic archipelagos: Hotspots for inferences of evolutionary process. Proc. Natl. Acad. Sci. USA.

[17] Howarth F.G., Mull W.P. (1992). Hawaiian Insects and Their Kin.

[18] Rubinoff D. (2008). Phylogeography and ecology of an endemic radiation of Hawaiian aquatic case-bearing moths (Hyposmocoma: Cosmopterigidae). Philos. Trans. R. Soc. B.

[19] Givnish T.J., Millam K.C., Mast A.R., Paterson T.B., Theim T.J., Hipp A.L., Henss J.M., Smith J.F., Wood K.R., Kenneth J.S. (2009). Origin, adaptive radiation and diversification of the Hawaiian lobeliads (Asterales: Campanulaceae). Proc. R. Soc. Lond. B Biol..

[20] Heed W.B. (1971). Host plant specificity and speciation in Hawaiian Drosophila. Taxon.

[21] Boake C.R.B. (2005). Sexual selection and speciation in Hawaiian Drosophila. Behav. Genet..

[22] Magnacca K.N., Foote D., O’Grady P.M. (2008). A review of the endemic Hawaiian Drosophilidae and their host plants. Zootaxa.

[23] Roderick G.K., Percy D.M., Tilmon K. (2008). Host plant use, diversification, and coevolution: Insights from remote oceanic islands. Specialization, Speciation, and Radiation. Evolutionary Biology of Herbivorous Insects.

[24] Bennett G.M., O’Grady P.M. (2012). Host-plants shape insect diversity: Phylogeny, origin, and species diversity of native Hawaiian leafhoppers (Cicadellidae: Nesophrosyne). Mol. Phylogenet. Evol..

[25] Zimmerman E.C. (1948). Insects of Hawaii Introduction.

[26] Ashlock P.D., Gagne W.C. (1983). A remarkable new micropterous-nysius species from the Aeolian Zone of Mauna-Kea, Hawaii Island (Hemiptera, Heteroptera, Lygaeidae). Int. J. Entomol..

[27] Eiben J.A., Rubinoff D. (2010). Life history and captive rearing of the Wekiu bug (*Nysius wekiuicola*, Lygaeidae), an alpine carnivore endemic to the Mauna Kea volcano of Hawaii. J. Insect Conserv..

[28] Hardy D.E. (1965). Insects of Hawaii: Diptera Cyclorrapha II.

[29] Montgomery S.L. (1983). Carnivorous caterpillars: The behavior, biogeography and conservation of Eupithecia (Lepidoptera: Geometridae) in the Hawaiian Islands. GeoJournal.

[30] O’Connor T.K., Humphrey P.T., Lapoint R.T., Whiteman N.K., O’Grady P.M. (2014). Microbial interactions and the ecology and evolution of Hawaiian Drosophilidae. Front. Microbial..

[31] Ort B.S., Bantay R.M., Pantoja N.A., O’Grady P.M. (2012). Fungal diversity associated with Hawaiian Drosophila host plants. PLoS ONE.

[32] Newell P.D., Douglas A.E. (2014). Interspecies interactions determine the impact of the gut microbiota on nutrient allocation in *Drosophila melanogaster*. Appl. Environ. Microbiol..

[33] Buchner P. (1965). Endosymbiosis of animals with plant microorganisms. Interscience.

[34] Houk E.J., Griffiths G.W. (1980). Intracellular symbiotes of the Homoptera. Annu. Rev. Entomol..

[35] Shigenobu S., Wilson A.C. (2011). Genomic revelations of a mutualism: The pea aphid and its obligate bacterial symbiont. Cell. Mol. Life Sci..

[36] Caporaso J.G., Lauber C.L., Walters W.A., Berg-Lyons D., Lozupone C.A., Turnbaugh P.J., Fierer N., Knight R. (2011). Global patterns of 16S rRNA diversity at a depth of millions of sequences per sample. Proc. Natl. Acad. Sci. USA.

[37] Gordon A., Hannon G.J. Fastx-Toolkit. FASTQ/A Short-Reads Preprocessing Tools. http://hannonlab.cshl.edu/fastx_toolkit/.

[38] Zhang J., Kobert K., Flouri T., Stamatakis A. (2013). PEAR: A fast and accurate Illumina Paired-End reAd mergeR. Bioinformatics.

[39] Edgar R.C., Haas B.J., Clemente J.C., Quince C., Knight R. (2011). UCHIME improves sensitivity and speed of chimera detection. Bioinformatics.

[40] Rognes T., Flouri T., Nichols B., Quince C., Mahé F. (2016). VSEARCH: A versatile open source tool for metagenomics. PeerJ.

[41] Caporaso J.G., Kuczynski J., Stombaugh J., Bittinger K., Bushman F.D., Costello E.K., Fierer N., Gonzalez Pena A., Goodrich J.K., Gordon J.I. (2010). QIIME allows analysis of high-throughput community sequencing data. Nat. Methods.

[42] Quast C., Pruesse E., Yilmaz P., Gerken J., Schweer T., Yarza P., Peplies J., Glöckner F.O. (2012). The SILVA ribosomal RNA gene database project: Improved data processing and web-based tools. Nucleic Acids Res..

[43] R Core Team R: A Language and Environment for Statistical Computing. R Foundation for Statistical Computing. http://www.R-project.org/.

[44] Oksanen J., Blanchet G., Friendly M., Kindt R., Legendre P., McGlinn D., Minchin P., O’Hara R., Simpson G., Solymos P. Vegan: Community Ecology Package. https://cran.r-project.org/web/packages/vegan/index.html/.

[45] Bray J.R., Curtis J.T. (1957). An ordination of upland forest communities of Southern Wisconsin. Ecol. Monogr..

[46] Bennett G.M., Moran N.A. (2013). Small, smaller, smallest: The origins and evolution of ancient dual symbioses in a phloem-feeding insect. Genome Biol. Evol..

[47] Matsuura Y., Kikuchi Y., Hosokawa T., Koga R., Meng X.Y., Kamagata Y., Nikoh N., Fukatsu T. (2012). Evolution of symbiotic organs and endosymbionts in lygaeid stinkbugs. ISME J..

[48] Engel P., Moran N.A. (2013). The gut microbiota of insects—Diversity in structure and function. FEMS Microbiol. Rev..

[49] Shapira M. (2016). Gut microbiotas and host evolution: Scaling up symbiosis. Trends Ecol. Evol..

[50] Hammer T.J., Janzen D.H., Hallwachs W., Jaffe S.L., Fierer N. (2017). Caterpillars lack a resident gut microbiome. Proc. Natl. Acad. Sci. USA.

[51] Douglas A.E. (2009). The microbial dimension in insect nutritional ecology. Funct Ecol.

[52] Liu N., Yan X., Zhang M., Xie L., Wang Q., Huang Y., Zhou X., Wang S., Zhou Z. (2011). Microbiome of fungus-growing termites: A new reservoir for lignocellulase genes. Appl. Environ. Microbiol..

[53] Weiss B., Aksoy S. (2011). Microbiome influences on insect host vector competence. Trends Parasitol..

[54] Ceja-Navarro J.A., Vega F.E., Karaoz U., Hao Z., Jenkins S., Lim H.C., Kosina P., Infante F., Northen T.R., Brodie E.L. (2015). Gut microbiota mediate caffeine detoxification in the primary insect pest of coffee. Nat. Commun..

[55] Biddle A., Stewart L., Blanchard J., Leschine S. (2013). Untangling the genetic basis of fibrolytic specialization by Lachnospiraceae and Ruminococcaceae in diverse gut communities. Diversity.

[56] Matthews K.A., Kaufman T.C., Gelbart W.M. (2005). Research resources for *Drosophila*: The expanding universe. Nat. Rev. Genet..

[57] Drysdale R. (2008). FlyBase: A database for the *Drosophila* research community. Methods Mol. Biol..

[58] Bellen H.J., Tong C., Tsuda H. (2010). 100 years of Drosophila research and its impact on vertebrate neuroscience: A history lesson for the future. Nat. Rev. Neurosci..

[59] Carson H.L., Hardy D.E., Spieth H.T., Stone W.S., Hecht M.K., Steere W.C. (1970). The evolutionary biology of the Hawaiian Drosophilidae. Essays in Evolution and Genetics in Honor of Theodosius Dobzhansky.

[60] Carson H.L., Kaneshiro K.Y. (1976). *Drosophila* of Hawaii: Systematics and ecological genetics. Annu. Rev. Ecol. Syst..

[61] Kambysellis M.P., Craddock E.M., Givnish T.J., Sytsma K.J. (1997). Ecological and reproductive shifts in the diversification of the endemic Hawaiian *Drosophila*. Molecular Evolution and Adaptive Radiation.

[62] Martinson V.G., Carpinteyro-Ponce J., Moran N.A., Markow T.A. (2017). A distinctive and host-restricted gut microbiota in populations of a cactophilic Drosophila species. Appl. Environ. Microbiol..

[63] Chandler J.A., Lang J.M., Bhatnagar S., Eisen J.A., Kopp A. (2011). Bacterial communities of diverse Drosophila species: Ecological context of a host–microbe model system. PLoS genet..

[64] Powell J.E., Martinson V.G., Urban-Mead K., Moran N.A. (2014). Routes of acquisition of the gut microbiota of the honey bee *Apis mellifera*. Appl. Environ. Microbiol..

[65] Pramono A.K., Sakamoto M., Iino T., Hongoh Y., Ohkuma M. (2015). *Dysgonomonas termitidis sp. nov*., isolated from the gut of the subterranean termite *Reticulitermes speratus*. Int. J. Syst. Evol. Microbiol..

[66] Brune A., Dietrich C. (2015). The gut microbiota of termites: Digesting the diversity in the light of ecology and evolution. Annu. Rev. Microbiol..

[67] Moran N.A., Tran P., Gerardo N.M. (2005). Symbiosis and Insect Diversification: An Ancient Symbiont of Sap-Feeding Insects from the Bacterial Phylum Bacteroidetes. Appl. Environ. Microbiol..

[68] Bressan A., Mulligan K.L. (2013). Localization and morphological variation of three bacteriome-inhabiting symbionts within a planthopper of the genus *Oliarus* (Hemiptera: Cixiidae). Environ. Microbiol. Rep..

[69] Sandström J., Moran N. (1999). How nutritionally imbalanced is phloem sap for aphids?. Entomol. Exp. Appl..

[70] McCutcheon J.P., Moran N.A. (2010). Functional convergence in reduced genomes of bacterial symbionts spanning 200 My of evolution. Genome Biol. Evolut..

[71] Bennett G.M., Moran N.A. (2015). Heritable symbiosis: The advantages and perils of an evolutionary rabbit hole. Proc. Natl. Acad. Sci. USA.

[72] Sailendharan S., Kost C., Kaltenpoth M. (2017). Symbiont Acquisition and Replacement as a Source of Ecological Innovation. Trends Microbiol..

[73] Toenshoff E.R., Gruber D., Horn M. (2012). Co-evolution and symbiont replacement shaped the symbiosis between adelgids (Hemiptera: Adelgidae) and their bacterial symbionts. Environ. Microbiol..

[74] Koga R., Bennett G.M., Cryan J.R., Moran N.A. (2013). Evolutionary replacement of obligate symbionts in an ancient and diverse insect lineage. Environ. Microbiol..

[75] Werren J.H., Baldo L., Clark M.E. (2008). Wolbachia: Master manipulators of invertebrate biology. Nat. Rev. Microbiol..

[76] Hilgenboecker K., Hammerstein P., Schlattmann P., Telschow A., Werren J.H. (2008). How many species are infected with Wolbachia? A statistical analysis of current data. FEMS Microbiol. Lett..

[77] Schneider D., Miller W.J., Riegler M., Zchori-Fein E., Bourtzis K. (2011). Arthropods shopping for Wolbachia. Manipulative Tenants: Bacteria Associated with Arthropods.

[78] Bennett G.M., Pantoja N.A., O’Grady P.M. (2012). Diversity and phylogenetic relationships of Wolbachia in Drosophila and other native Hawaiian insects. Fly.

[79] O’Connor L., Plichart C., Sang A.C., Brelsfoard C.L., Bossin H.C., Dobson S.L. (2012). Open release of male mosquitoes infected with a Wolbachia biopesticide: Field performance and infection containment. PLoS Negl. Trop. Dis..

[80] Blaxter M.L. (2004). The promise of a DNA taxonomy. Philos. Trans. R. Soc. B.

[81] Schoch C.L., Seifert K.A., Huhndorf S., Robert V., Spouge J.L., Levesque C.A., Chen W. (2012). Fungal Barcoding Consortium. Nuclear ribosomal internal transcribed spacer (ITS) region as a universal DNA barcode marker for Fungi. Proc. Natl. Acad. Sci. USA.

